# Role of Omega-3 Fatty Acids in IgA Nephropathy: An Updated Review of Mechanisms and Evidence

**DOI:** 10.3390/jcm15166332

**Published:** 2026-08-16

**Authors:** Hulya Taskapan, Luxcia Kugathasan, Labib Faruque, Tabo Sikaneta, Paul Tam

**Affiliations:** 1 Division of Nephrology, Department of Medicine, Toronto General Hospital Research Institute, University Health Network, Toronto, ON M5G 2C4, Canada; 2 Temerty Faculty of Medicine, University of Toronto, Toronto, ON M5S 3K9, Canada; 3 Department of Nephrology, Scarborough Health Network, Toronto, ON M1P 2V5, Canada; 4 Department of Family and Community Medicine, University of Toronto, Toronto, ON M5S 1A1, Canada

**Keywords:** IgA nephropathy, omega-3 fatty acids, EPA, DHA, fish oil, proteinuria, renal protection, inflammation, clinical trials, meta-analysis

## Abstract

**Introduction**: IgA nephropathy (IgAN) is a leading cause of end-stage renal disease. Given the significant adverse effects and inconsistent long-term efficacy of conventional immunosuppressive strategies, there is an unmet need for safer adjunctive therapies. Omega-3 polyunsaturated fatty acids (PUFAs) have been proposed as potential candidates to address this therapeutic gap. **Purpose**: This narrative review summarizes the proposed mechanisms of action of omega-3 PUFAs in IgAN and critically evaluates the current clinical evidence regarding their therapeutic potential and limitations. **Mechanisms**: Emerging experimental data suggest that omega-3 PUFAs may modulate inflammatory and fibrotic pathways relevant to kidney injury. Proposed mechanisms include modulation of eicosanoid metabolism, attenuation of NLR family pyrin domain-containing 3 (NLRP3) inflammasome activation, and generation of specialized pro-resolving mediators. In experimental studies, omega-3 PUFAs may also suppress nuclear factor kappa B (NF-κB)-driven transcription and attenuate mesangial cell proliferation, IgA immune-complex deposition, and transforming growth factor beta 1 (TGF-β1)/Smad3-mediated fibrotic signaling. **Clinical Evidence: Conclusions**: Omega-3 PUFAs have biological plausibility as adjunctive therapy in IgAN, but their clinical benefit remains uncertain. Available randomized trials and meta-analyses suggest possible modest effects on proteinuria in some settings, whereas evidence for preservation of kidney function or prevention of kidney failure is inconsistent and of low certainty. Future well-designed trials incorporating guideline-directed background therapy and biomarker-guided patient selection are essential to determine optimal dosing, formulation, biological exposure, and whether any patient subgroups derive clinically meaningful benefit.

## 1. Introduction

IgA nephropathy (IgAN) is the most common primary glomerulonephritis worldwide and a leading cause of chronic kidney disease (CKD) and kidney failure [1]. Approximately 20–40% of affected individuals progress to ESRD within 10–20 years of diagnosis, which accounts for a substantial proportion of the global dialysis population [1]. Despite this significant disease burden, the clinical course remains highly variable, ranging from persistent low-grade proteinuria to rapidly progressive kidney failure.

The molecular pathogenesis of IgA nephropathy is commonly described by a multi-hit model involving increased production of galactose-deficient IgA1, formation of antiglycan autoantibodies, generation of circulating immune complexes, mesangial deposition, and downstream glomerular injury [2,3,4,5,6,7,8]. Hit 1 involves increased production of galactose-deficient IgA1 (Gd-IgA1), driven by dysregulated mucosal immunity, altered B-cell responses, and abnormal O-glycosylation in the IgA1 hinge region [4,6,7]. These aberrantly glycosylated IgA1 molecules expose terminal GalNAc-containing epitopes that are recognized by antiglycan autoantibodies, predominantly of the IgG and IgA isotypes (Hit 2) [6,9,10]. The resulting Gd-IgA1-containing immune complexes (Hit 3) accumulate in the mesangium, potentially involving interactions with transferrin receptor 1 (CD71) and soluble CD89 (sCD89)-related pathways [2,3,4,5].

Mesangial deposition triggers Hit 4, characterized by mesangial cell activation, inflammatory mediator release, and complement activation. Cytokines such as IL-6 and TNF-α promote local inflammation and extracellular matrix expansion, while complement activation through the alternative and lectin pathways contributes to progressive kidney injury [5,7,8,11,12]. Activation of the alternative, lectin, and terminal complement pathways contributes to glomerular inflammation and kidney injury in IgAN. Glomerular C5b-9 deposition and complement activation products, including C3a and C5a, have been associated with proteinuria, histologic injury, and disease progression; however, the precise causal contribution of individual complement fragments to podocyte, endothelial, and tubulointerstitial injury remains incompletely defined [11,12,13,14].

Beyond the classical multi-hit model, recent studies suggest that IgAN may involve additional pathogenic processes, including recurrent mucosal immune activation, persistent intrarenal immune dysregulation, and direct interactions between mesangial cells and pathogenic autoantibodies [15,16]. Recurrent mucosal immune activation, gut microbiome alterations, and persistent intrarenal inflammation may contribute to ongoing kidney injury and disease progression. Persistent complement activation and profibrotic pathways, including TGF-β/Smad3 signaling, may further drive extracellular matrix accumulation and nephron loss [17]. These pathogenic processes provide a biological rationale for interventions that may modulate intrarenal inflammation, lipid mediators, and fibrotic signaling. However, modulation of these downstream pathways would not necessarily correct the upstream abnormalities in Gd-IgA1 production or anti-glycan autoantibody formation.

Current treatment is directed primarily at slowing disease progression, reducing proteinuria, and mitigating cardiovascular risk [1]. Optimized supportive care, including renin–angiotensin system (RAS) blockade and sodium–glucose cotransporter-2 (SGLT2) inhibitors, constitutes the foundation of IgA nephropathy management, while corticosteroids and other immunosuppressive agents are reserved for selected high-risk patients. However, these immunosuppressive therapies carry a considerable burden of adverse events, including infections, metabolic disturbances, and bone loss, and their long-term benefit–risk profile remains subject to ongoing debate [1]. There is therefore a recognized need for safer and well-tolerated adjunctive therapies.

Omega-3 polyunsaturated fatty acids (PUFAs), principally eicosapentaenoic acid (EPA) and docosahexaenoic acid (DHA), are derived from marine and fish oils and possess well-documented anti-inflammatory and immunomodulatory properties [18,19,20]. Several pathological mechanisms postulated in IgAN are amenable to modulation by these agents [7,8,21,22,23,24]. Across clinical studies, omega-3 PUFA supplementation has generally been well tolerated, with mild gastrointestinal symptoms among the most frequently reported adverse effects [25,26,27]. Their favorable tolerability profile and established cardiovascular benefits in CKD populations [28] may further support their candidacy for a safer adjunctive role in IgAN management. This review summarizes the proposed mechanisms of action of omega-3 PUFAs in IgAN and critically evaluates the current clinical evidence regarding their therapeutic potential and limitations.

## 2. Proposed Mechanisms of Action of Omega-3 PUFAs in IgAN

**Figure 1:**
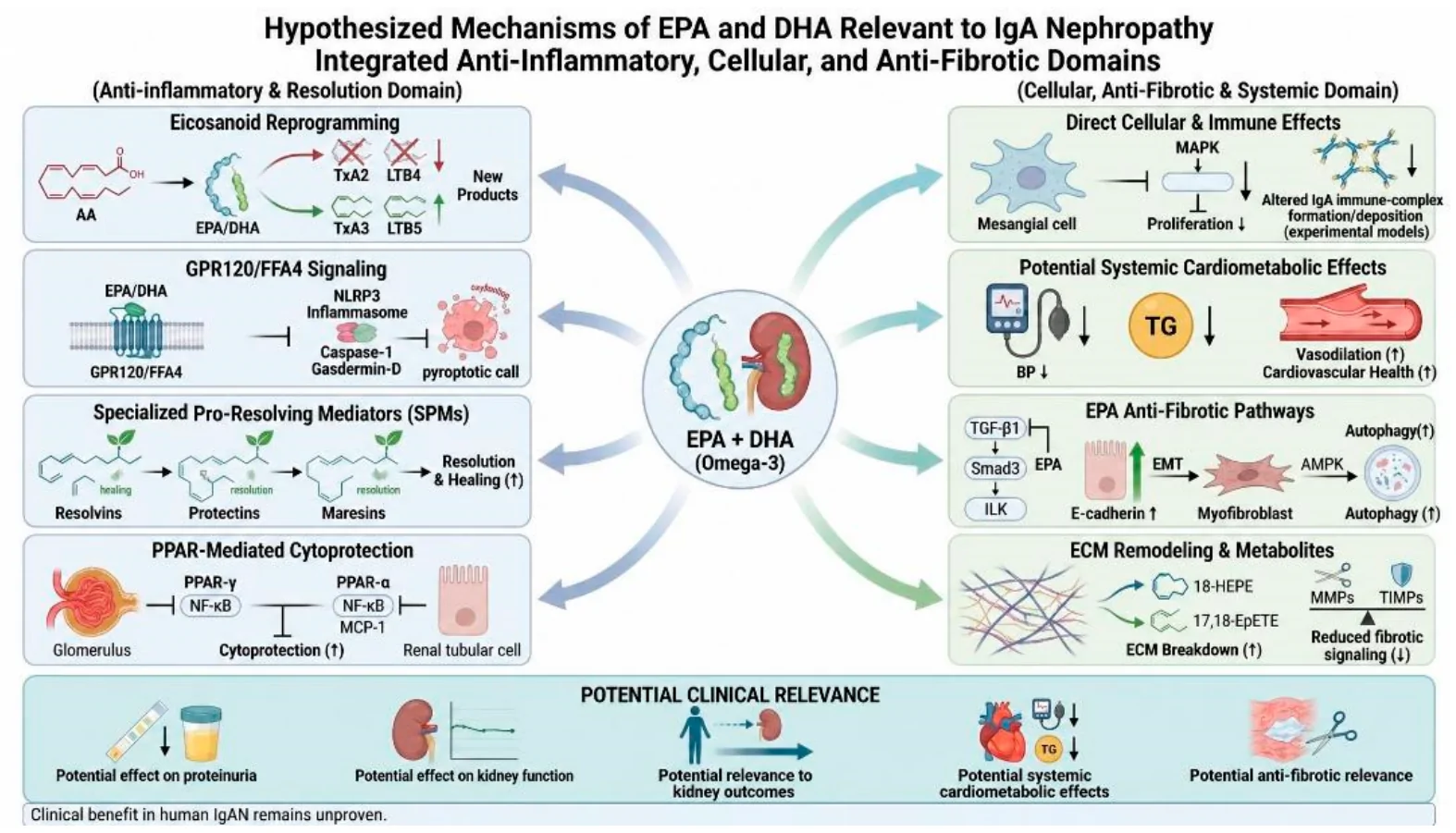
Proposed mechanisms through which EPA and DHA may influence inflammatory and fibrotic pathways relevant to IgA nephropathy. The pathways and clinical outcome labels shown are conceptual and are derived predominantly from in vitro and preclinical studies, including experimental IgAN and non-IgAN kidney disease models. Labels describing potential effects on proteinuria, kidney function, kidney outcomes, cardiometabolic effects, and fibrosis represent potential areas of clinical relevance and should not be interpreted as established clinical benefits of omega-3 PUFAs in human IgAN. EPA and DHA may influence inflammatory and fibrotic pathways through three proposed axes. The Anti-Inflammatory & Resolution Domain (**left**) involves eicosanoid reprogramming, GPR120/FFA4-associated attenuation of NLRP3 inflammasome activation, and activation of specialized pro-resolving mediators (SPMs) and PPAR-signaling. The Cellular, Anti-Fibrotic & Systemic Domain (**right**) highlights experimental effects on mesangial-cell proliferation and IgA-related immune-complex formation or deposition, alongside systemic cardiometabolic benefits and anti-fibrotic pathways targeting TGF-β1/Smad3, EMT, and autophagic flux. These pathways represent mechanistic hypotheses derived predominantly from in vitro and preclinical studies. Their relationship to clinical outcomes in human IgAN remains unproven. Abbreviations: AA, arachidonic acid; AMPK, AMP-activated protein kinase; BP, blood pressure; DHA, docosahexaenoic acid; ECM, extracellular matrix; EMT, epithelial–mesenchymal transition; EPA, eicosapentaenoic acid; GPR, G-protein-coupled receptor; ILK, integrin-linked kinase; LTB, leukotriene B; MAPK, mitogen-activated protein kinase; MCP-1, monocyte chemoattractant protein-1; MMPs, matrix metalloproteinases; NF-κB, nuclear factor kappa B; NLRP3, NLR family pyrin domain-containing 3 (NLRP3); PPAR, peroxisome proliferator-activated receptor; Smad3, mothers against decapentaplegic homolog 3; SPMs, specialized pro-resolving mediators; TG, triglycerides; TGF-β1, transforming growth factor beta 1; TIMPs, tissue inhibitors of metalloproteinases; TxA, thromboxane A; upward arrows (↑) indicate an increase or activation, downward arrows (↓) indicate a decrease or inhibition, and the dagger symbol. Colors are used for visual distinction between different mechanistic domains.

Omega-3 PUFAs exert a broad range of anti-inflammatory and immunomodulatory effects through multiple pathways, some of which have been shown in preclinical models to interact with the pathological processes driving glomerular injury in IgAN [18,19,20]. These mechanisms are organized below according to their principal site and mode of action and are summarized schematically in Figure 1.

### 2.1. Eicosanoid Modulation: Redirecting the Inflammatory Lipid Mediator Profile

The most established mechanism through which omega-3 PUFAs exert anti-inflammatory effects is the competitive modulation of eicosanoid biosynthesis. Upon intake, EPA and DHA are incorporated into cellular phospholipid bilayers, where they displace arachidonic acid (AA) as the preferred substrate for cyclooxygenase and lipoxygenase enzymes [18,19,29,30]. This substrate shift redirects production away from AA-derived mediators, such as thromboxane A2 (TxA2) and leukotriene B4 (LTB4), toward less pro-inflammatory derivatives, including thromboxane A3 (TxA3), prostacyclin I3 (PGI3), and leukotriene B5 (LTB5) [30] (Figure 1).

In the context of IgAN, where glomerular mesangial cells exhibit dysregulated eicosanoid metabolism leading to leukocyte recruitment and matrix expansion, this reprogramming provides a potentially relevant anti-inflammatory effect [22,23]. While systemic reductions in C-reactive protein (CRP), interleukin-6 (IL-6), and tumor necrosis factor-alpha (TNF-α) have been reported in some studies, these findings are not universally observed across clinical cohorts [18,19,29,30]. This inconsistency suggests that omega-3-mediated benefits in IgAN may be more localized to the kidney tissue microenvironment rather than reflected in systemic inflammatory markers.

### 2.2. Active Inflammation Resolution: Specialized Pro-Resolving Mediator Induction and Inflammasome/Pyroptosis Suppression

Beyond modulating eicosanoid production, omega-3 PUFAs actively promote the resolution phase of inflammation, a distinct biological process rather than passive decay [31,32,33]. EPA and DHA serve as precursors for specialized pro-resolving mediators (SPMs), including resolvins, protectins, and maresins, which help terminate inflammatory responses by promoting neutrophil clearance and macrophage efferocytosis, the process by which macrophages engulf and remove dead or dying cells [31,32,33]. The biosynthesis of these mediators may also be modulated by agents such as aspirin and statins [34]. This mechanistic observation does not support the routine use of aspirin or statins specifically to enhance omega-3 therapy in IgAN. Statins and antiplatelet therapy should be prescribed according to standard lipid-management and cardiovascular indications, with individual assessment of bleeding risk, kidney function, and concomitant medications. In addition to SPM production, omega-3 PUFAs may signal through G-protein-coupled receptor 120, also known as free fatty acid receptor 4 (GPR120/FFA4). Through β-arrestin-2 signaling, this pathway may suppress the assembly of the NOD-, LRR- and pyrin domain-containing protein 3 (NLRP3) inflammasome, a key node in IgAN-associated mesangial activation. By inhibiting this nodal protein complex, omega-3 PUFAs attenuate caspase-1 activity and the subsequent maturation of interleukin-1 (IL-1) [35,36,37,38].

This regulatory cascade further prevents pyroptosis, a highly inflammatory form of programmed cell death driven by gasdermin-D (GSDMD) cleavage. Experimental models of kidney injury indicate that GPR120 activation effectively suppresses the full pyroptotic cascade, including the reduction in cleaved GSDMD and caspase-1 [39]. Although this mechanism could offer a distinct layer of glomerular protection relevant to IgA immune complex deposition, these data remain limited to preclinical settings. Caution is therefore warranted when extrapolating these findings to human IgAN.

### 2.3. PPAR/NF-κB Signaling: Transcriptional Cytoprotection in Glomerular and Tubular Cells

A third, transcriptionally mediated layer of renoprotection involves modulation of peroxisome proliferator-activated receptor (PPAR) and nuclear factor kappa B (NF-κB) signaling in glomerular endothelial cells and proximal tubular epithelial cells, two key cellular targets in IgAN-associated kidney injury.

In glomerular endothelial cells, oxidized EPA activates PPAR-γ, thereby suppressing cytokine-induced leukocyte adhesion and transendothelial migration. This PPAR-γ engagement also inhibits NF-κB-driven expression of monocyte chemoattractant protein-1 (MCP-1), a chemokine with a well-established role in glomerular macrophage infiltration [40]. In proximal tubular epithelial cells, EPA and DHA attenuated lipopolysaccharide-induced NF-κB activation and MCP-1 expression through a PPAR-γ-dependent mechanism in HK-2 cells [41]. These findings suggest that omega-3 PUFAs may modulate PPAR-γ-associated inflammatory signaling in both glomerular endothelial and tubular epithelial cell models. However, these observations are derived from in vitro studies, and their relevance to kidney outcomes in human IgAN remains uncertain.

### 2.4. Direct Modulation of Mesangial Proliferation and IgA Immune Complex Deposition

Omega-3 PUFAs also directly modulate the pathological processes most specific to IgAN: mesangial cell proliferation and aberrant IgA immune complex deposition.

Preclinical studies, including in vitro and animal models of mesangial proliferative glomerulonephritis, have shown that fish oil supplementation reduces proteinuria, mesangial cell and matrix expansion, and glomerular cell proliferation [23]. The molecular basis for these effects has been suggested by in vitro studies, where DHA demonstrated the potential to selectively inhibit mesangial cell proliferation. This was modulated by MAPK signaling and cell cycle regulatory elements, specifically through downregulation of ERK activity, upregulation of JNK activity, inhibition of cyclin E kinase activity, and induction of the cyclin-dependent kinase inhibitor p21 [42]. These coordinated cell cycle effects suggest that DHA may inhibit the mesangial expansion that drives progressive glomerulosclerosis in IgAN (Figure 1).

Beyond mesangial proliferation, DHA has been shown to reduce circulating IgA concentrations, immune complex formation, and renal IgA deposition in a murine model of IgAN through suppression of splenic IL-6 and COX-2 mRNA expression and inhibition of p38, ERK1/2, and JNK1/2 MAPK phosphorylation [24]. These findings, derived from a murine model, suggest that DHA may influence immune processes relevant to IgA production and immune-complex formation in experimental disease. However, direct effects on Gd-IgA1 production, antiglycan autoantibody formation, or pathogenic immune-complex generation have not been established in human IgAN.

### 2.5. Systemic and Cardiometabolic Effects: Relevance to CKD Management in IgAN

Omega-3 PUFAs confer systemic benefits of direct relevance to IgAN-associated CKD, where cardiovascular disease constitutes a major source of morbidity and mortality independent of kidney outcomes [1]. Recent clinical evidence supports a reduction in major cardiovascular events with fish oil supplementation in high-risk CKD populations receiving hemodialysis [28]. However, evidence from hemodialysis cohorts cannot be directly extrapolated to patients with earlier-stage CKD or IgAN. Omega-3 PUFAs reduce serum triglyceride levels by enhancing lipoprotein lipase activity, a clinically meaningful effect given the recognized contribution of dyslipidemia to both IgAN progression and elevated cardiovascular risk in this patient group [43]. Additional systemic benefits include attenuation of platelet aggregation, promotion of vasorelaxation, and modest blood pressure reduction [44].

### 2.6. Anti-Fibrotic Effects: Suppression of the TGF-β1/Smad3 Pathway and Epithelial–Mesenchymal Transition

Tubulointerstitial fibrosis remains an important therapeutic challenge in IgAN and a target incompletely addressed by current therapies. Tubulointerstitial fibrosis is an important determinant of long-term kidney prognosis in IgAN; however, whether omega-3 PUFAs modify fibrotic progression in patients remains unknown. Current evidence for anti-fibrotic effects of EPA and DHA is derived predominantly from in vitro and preclinical models rather than from randomized clinical trials in IgAN.

Emerging in vitro experimental evidence suggests that EPA may modulate the TGF-β1/Smad3 signaling axis to exert anti-fibrotic effects in renal tubular cells. In albumin-induced epithelial–mesenchymal transition (EMT) in HK-2 proximal tubular cells, EPA significantly reduced protein levels of TGF-β1, integrin-linked kinase (ILK), and phosphorylated Smad3 [45]. These signaling changes were accompanied by suppression of mesenchymal markers (α-SMA, N-cadherin, vimentin) and restoration of the epithelial marker E-cadherin. EPA additionally remodeled the extracellular matrix (ECM) balance by upregulating matrix metalloproteinases MMP-1, MMP-3, and MMP-9 while downregulating tissue inhibitors of metalloproteinases TIMP-1 and TIMP-2, collectively favoring ECM degradation over accumulation [45] (Figure 1).

Mechanistic studies have identified CYP450-dependent EPA metabolites, including 18-HEPE, 17,18-EpETE, and 17,18-diHETE, as potential autonomous mediators of anti-fibrotic activity in renal fibroblasts, with their inhibitory effects on TGF-β1-stimulated α-SMA expression substantially reversed by CYP inhibition [46]. In addition, both EPA and DHA have been shown to attenuate TGF-β1-induced epithelial-to-mesenchymal transition (EMT) through AMPK-mediated restoration of autophagic flux in tubular cells [47]. Together, these experimental findings generate the hypothesis that omega-3 PUFAs may influence pathways involved in tubular injury and fibrotic remodeling. However, whether modulation of these pathways translates into clinically meaningful preservation of kidney function, reduction in fibrosis, or improved renal survival in patients with IgAN has not been established. Although similar inflammatory and fibrotic pathways are implicated in other etiologies of CKD, extrapolation of these experimental findings beyond IgAN is speculative and may not be interpreted as evidence for clinical benefit across CKD populations.

**Figure 2:**
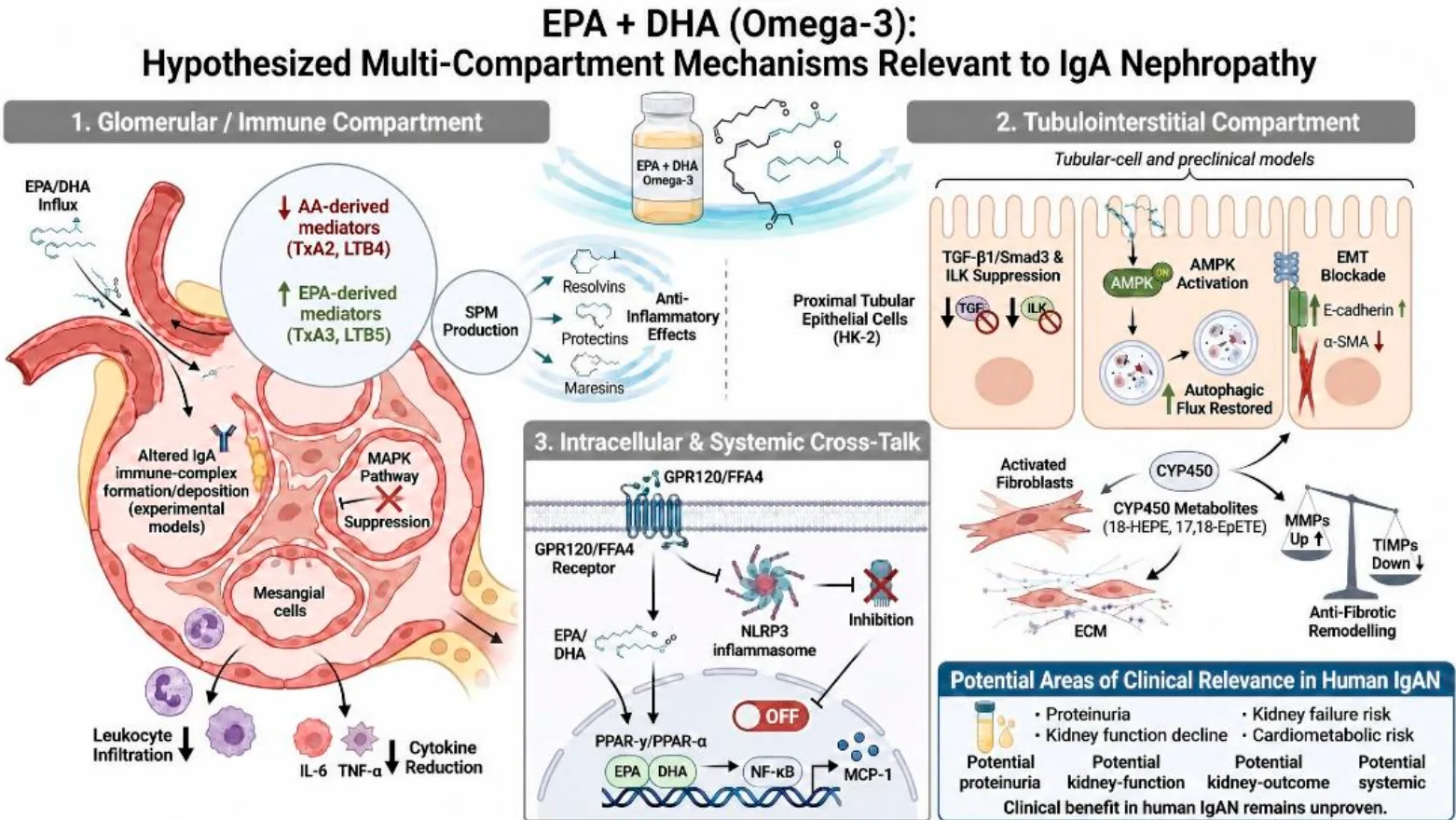
Hypothesized multi-compartment mechanisms through which EPA and DHA may influence inflammatory and fibrotic pathways relevant to IgA nephropathy. The pathways shown are derived predominantly from in vitro and preclinical studies, including experimental IgAN and non-IgAN kidney disease models. Their relationship to proteinuria, eGFR decline, kidney failure, and renal survival in human IgAN remains unproven. (1) Glomerular and immune compartment: In experimental models, EPA and DHA may alter eicosanoid substrate availability, promote specialized pro-resolving mediator biosynthesis, and modulate MAPK-associated signaling. These effects have been associated with reduced mesangial-cell proliferation and altered IgA immune-complex formation or deposition in selected experimental systems. (2) Tubulointerstitial compartment: In cellular and preclinical models, EPA and DHA may modulate TGF-β1/Smad3 and ILK signaling, AMPK-associated autophagic flux, epithelial-to-mesenchymal transition, and extracellular-matrix remodeling. (3) Intracellular and systemic cross-talk: Omega-3 PUFAs may activate GPR120/FFA4-associated signaling and attenuate NLRP3 inflammasome activation. EPA and DHA may also modulate PPAR-dependent pathways, including PPAR-γ-associated signaling, with downstream attenuation of NF-κB-mediated inflammatory transcription. These mechanisms are derived predominantly from in vitro and preclinical studies. Their relationship to proteinuria, eGFR decline, kidney failure, or renal survival in human IgAN remains unproven. Abbreviations: AMPK, AMP-activated protein kinase; CYP450, cytochrome P450; DHA, docosahexaenoic acid; ECM, extracellular matrix; EMT, epithelial–mesenchymal transition; EPA, eicosapentaenoic acid; FFA4, free fatty acid receptor 4 (GPR120); IL-6, interleukin-6; ILK, integrin-linked kinase; LTB, leukotriene B; MAPK, mitogen-activated protein kinase; MCP-1, monocyte chemoattractant protein-1; MMPs, matrix metalloproteinases; NF-κB, nuclear factor kappa B; NLRP3, NLR family pyrin domain-containing 3 (NLRP3); PPAR, peroxisome proliferator-activated receptor; Smad3, mothers against decapentaplegic homolog 3; SPMs, specialized pro-resolving mediators; TGF-β1, transforming growth factor beta 1; TIMPs, tissue inhibitors of metalloproteinases; TNF-α, tumor necrosis factor-alpha; TxA, thromboxane A; the red cross mark (×) indicates inhibition; upward arrows (↑) denote increase/activation, and downward arrows (↓) denote decrease/inhibition. Colors are used for visual distinction between different mechanistic domains.

Taken together, these cellular and preclinical observations provide a biologically plausible rationale for further evaluation of omega-3 PUFAs in IgAN. Whether these mechanistic effects persist in humans receiving contemporary guideline-directed supportive care and targeted disease-modifying therapies remains uncertain. The integrated cellular, molecular, and proposed multi-compartment pathways of EPA and DHA across the glomerular, tubulointerstitial, and systemic domains are depicted in Figure 2.

## 3. Omega-3 PUFAs in the Contemporary Therapeutic Landscape of IgA Nephropathy

Contemporary management of IgA nephropathy is based on risk stratification and a stepwise approach that prioritizes reduction in proteinuria, preservation of kidney function, management of hypertension, and minimization of treatment-related toxicity [1,48]. Optimized supportive care remains the foundation of management for all patients. This includes lifestyle and dietary measures, particularly sodium restriction, smoking cessation, weight management where appropriate, cardiovascular risk reduction, and maximally tolerated renin–angiotensin system blockade with an angiotensin-converting enzyme inhibitor or angiotensin receptor blocker (RAS blockade) in patients with proteinuria and/or hypertension [1,48]. Sodium–glucose cotransporter-2 inhibitors are also increasingly incorporated into supportive care for eligible patients with chronic kidney disease, including those with IgAN, based on evidence of reduced proteinuria and slower progression of kidney disease [1,49].

For patients who remain at increased risk of progression despite optimized supportive care, disease-modifying treatment may be considered according to disease severity, kidney function, comorbidities, access, and local regulatory approval. Available options differ across jurisdictions. Systemic glucocorticoids may be considered in carefully selected patients who remain at high risk of progression despite optimized supportive care, particularly those with persistent proteinuria, after assessment of kidney function, comorbidities, and treatment-related infectious and metabolic risks [1,48]. Targeted-release budesonide, complement-directed therapies, and B-cell/APRIL-directed therapies may be considered according to individual risk, eligibility criteria, kidney function, local regulatory approval, and availability [1,50,51,52,53,54,55,56,57]. These therapies are not indicated for all patients with IgAN, particularly those with low-risk disease, stable kidney function, or substantial chronic irreversible kidney damage [1].

Targeted-release budesonide, which is designed to act at the level of ileal Peyer’s patches and thereby modulate mucosal immune pathways relevant to Gd-IgA1 production, has demonstrated sustained antiproteinuric effects and preservation of kidney function in randomized trials [55,56]. Sparsentan, a dual endothelin type A and angiotensin II type 1 receptor antagonist, provides an additional non-immunosuppressive approach to proteinuria reduction and kidney protection in appropriate patients [57]. Alternative complement pathway inhibition with iptacopan has also shown favorable effects on proteinuria and kidney outcomes in clinical trials; however, its availability and approved indication vary by jurisdiction [50,51].

A number of therapies targeting upstream immune mechanisms are emerging but remain investigational or have variable regulatory status internationally. These include APRIL-directed therapies, B-cell-directed therapies, and agents targeting the BAFF/APRIL pathway. Early-phase studies have reported reductions in proteinuria and changes in disease-related biomarkers; however, long-term kidney outcomes, optimal sequencing, comparative effectiveness, and regulatory status remain uncertain [52,53,54]. These agents are intended to interfere more directly with pathogenic processes involved in IgAN, including abnormal mucosal immune activation, B-cell survival, Gd-IgA1 production, autoantibody formation, and complement-mediated glomerular injury. Their precise role in treatment algorithms will depend on confirmation of long-term efficacy, safety, accessibility, and regulatory approval [1,50,51,52,53,54].

Within this contemporary treatment landscape, omega-3 PUFAs are not considered a substitute for optimized supportive care or for approved disease-modifying therapies where indicated. Rather, if used at all, omega-3 PUFAs should be regarded as an individualized and clinically unproven adjunctive intervention. Their potential incremental benefit when used alongside contemporary therapy has not been established. Contemporary therapy may include maximally tolerated RAS blockade or sparsentan, as appropriate, together with SGLT2 inhibition and, where indicated, targeted-release budesonide, complement-directed therapy, or B-cell-directed therapy. Importantly, most omega-3 PUFA trials in IgAN predated the widespread use of contemporary therapeutic approaches [1,25,26,27,48,49]. Given the inconsistency and low certainty of the available evidence, the Kidney Disease: Improving Global Outcomes (KDIGO) 2025 guideline does not recommend fish oil as routine therapy for IgAN [1]. This guidance does not imply that omega-3 supplementation is an established disease-modifying therapy.

## 4. Clinical Evidence

### 4.1. Overview: A Historically Variable Evidence Base

Clinical investigation of omega-3 PUFAs in IgAN spans more than four decades, evolving from small uncontrolled observations to randomized trials and systematic meta-analytic syntheses. A summary of key trials is provided in Table 1.

### 4.2. Early Studies: Initial Signals and Inconsistencies

Among the earliest reports in the field, Hamazaki et al. [58] reported improved renal function in a small cohort of patients with IgAN (N = 20; 10 receiving EPA [1.6 g daily] plus DHA [1.0 g daily] and 10 untreated controls) over a one-year observation period. No patients in the treated group required hemodialysis compared with two patients in the control group. In parallel, essential fatty acid deficiency profiles were identified in patients with IgAN [22], raising the hypothesis that supplementation might correct lipid imbalances relevant to disease activity.

However, these initial positive signals were not consistently reproduced in subsequent prospective studies. A two-year prospective trial of high-dose EPA supplementation (10 g/day, a substantially higher dose than that used by Hamazaki et al.) reported no meaningful improvement in kidney function despite confirmed alterations in plasma fatty acid profiles [59], suggesting that shifts in lipid composition alone are insufficient to confer renoprotection. A double-blind, randomized, placebo-controlled trial evaluating omega-3 PUFAs (EPA 3.3 g + DHA 1.8 g daily) also showed no significant effects on proteinuria or urinary red blood cell counts. However, it notably revealed a slight but statistically significant eGFR reduction from baseline only in the active treatment group and not in the corn oil control [26], prompting early concerns over potential adverse renal hemodynamic effects at high doses.

These early studies were limited by small sample sizes, variable randomization methods, and, notably, the absence of background RAS blockade, now a standard of care in IgAN. All of these limitations may have contributed to the heterogeneity of results.

### 4.3. Evidence from a Major Randomized Trial and Its Long-Term Follow-Up

A pivotal advancement came with the multicenter, randomized, placebo-controlled trial by Donadio et al. [27], which remains the most influential single trial of omega-3 PUFAs in IgAN to date. Patients with proteinuric IgAN were randomized to receive 12 g/day of fish oil or olive-oil placebo for 2 years. The fish-oil preparation provided approximately 1.7–1.9 g/day of EPA and 1.0–1.4 g/day of DHA, depending on the formulation used during the study [27]. The primary endpoint was a ≥50% increase in serum creatinine during the treatment period. This endpoint occurred in 6% of patients assigned to fish oil and 33% assigned to placebo (*p* = 0.002). Proteinuria was slightly reduced in the fish-oil group, although the between-group difference was not statistically significant. During extended follow-up, the cumulative incidence of death or end-stage renal disease was 10% in the fish-oil group and 40% in the placebo group at 4 years (*p* = 0.006) [27].

Extended follow-up of this same cohort over a median of 6.4 years showed that early and sustained fish oil treatment continued to slow kidney disease progression in high-risk patients [25].

Several methodological considerations regarding this trial merit discussion. First, the choice of olive oil as a placebo warrants scrutiny: olive oil is not pharmacologically inert and contains constituents with recognized anti-inflammatory properties. If anything, this would likely have blunted rather than exaggerated the observed treatment effect, but it nonetheless complicates interpretation of the control condition. Second, the event rate in the placebo group, 33% reaching the primary endpoint at two years and 40% developing ESRD or all-cause mortality by four years, has been noted as unusually high, raising the possibility that the treatment effect was magnified by unexpectedly rapid progression in the control arm, a point acknowledged by the authors themselves [25] and further highlighted in recent critical systematic reviews evaluating trial heterogeneity [67,68,69,70,71]. Third, external validity is limited by the modest sample size (n = 106) and the concentration of nearly half of participants at a single center, despite the trial’s multicenter design [27]. These considerations do not negate the trial’s contribution, but they contextualize the strength of its conclusions and have informed the design of subsequent studies with larger sample size and robust methods. The extended follow-up was observational after completion of the randomized treatment period; therefore, its findings should not be interpreted as providing the same level of causal certainty as the original randomized comparison.

### 4.4. Dose–Response Considerations

Following the landmark trial, questions arose regarding the dose required to influence kidney outcomes. A randomized comparison of high-dose (EPA 3.76 g + DHA 2.94 g daily) versus low-dose (EPA 1.88 g + DHA 1.47 g daily) omega-3 PUFAs in 73 patients with severe IgAN demonstrated similar efficacy between the two regimens over 2 years: the annualized median increase in serum creatinine was 0.08 mg/dL in the low-dose group and 0.10 mg/dL in the high-dose group (*p* = 0.51), and the number of patients progressing to ESRD did not differ significantly (10 vs. 8, *p* = 0.56) [60]. Both regimens slowed renal function loss relative to historical placebo-treated controls. The study did not identify a clear dose–response relationship; however, the absence of a placebo-controlled group limits inference regarding the efficacy of either regimen.

A subsequent randomized controlled trial tested a very low-dose regimen (EPA 0.85 g + DHA 0.57 g daily) in 14 high-risk patients versus 14 untreated controls over four years [61]. In the active treatment group, only 1 of 14 patients (7%) reached the primary endpoint of a ≥50% increase in serum creatinine (or a ≥50% decline in GFR), compared with 6 of 14 controls (43%; p<0.01). Notably, the mean annual decline in eGFR was significantly slower in the treated group compared to controls (−1.4 vs. −3.0 mL/min/year; p<0.001), parallel to a lower mean annual increase in serum creatinine (0.2 vs. 1.0 mg/dL; p<0.01) [61]. While these long-term renal outcomes are promising, the small sample size warrants caution in drawing definitive conclusions.

Collectively, these small historical studies generated hypotheses regarding dose and potential kidney effects; however, they do not establish a dose–response relationship or confirm clinically meaningful renoprotection.

### 4.5. Beyond Glomerular Filtration: Vascular, Tubular, and Combination Strategies

Evidence that omega-3 PUFAs may confer renoprotection through effects not captured by eGFR alone comes from a single-center study by Sulikowska et al. [62]. In 20 patients with IgAN treated with EPA (0.81 g) plus DHA (0.54 g) daily for 12 months, dopamine-induced glomerular filtration response, a measure of renal functional reserve, increased from 14.9% to 30.3% (*p* < 0.01), while urinary N-acetyl-β-D-glucosaminidase (NAG), a sensitive biomarker of proximal tubular injury, declined from 8.3 to 6.0 U/g creatinine (*p* < 0.01). Proteinuria and serum homocysteine levels also decreased significantly, yet creatinine clearance remained unchanged. These exploratory findings raise the hypothesis that omega-3 PUFAs may influence markers of renal functional reserve or tubular injury that are not reflected by conventional filtration measures. However, the uncontrolled design, small sample size, and use of surrogate physiological markers preclude conclusions regarding clinically meaningful renoprotection.

### 4.6. Combination with Ras Blockade

Several studies have explored the addition of omega-3 PUFAs to established background therapy. In a randomized controlled trial, the addition of omega-3 PUFAs (3 g/day) to RAS blockade reduced proteinuria by 72.9% from baseline, whereas no significant reduction was observed with RAS blockade alone [64]. A significant decrease in hematuria was also observed in the omega-3 group. In a separate study, the addition of EPA (0.9–1.8 g/day) to RAS inhibition was associated with a more rapid antiproteinuric response compared with RAS inhibition combined with dilazep, an antiplatelet and vasodilator agent [65]. This small study reported a more rapid reduction in proteinuria in the EPA-containing regimen; however, the comparator, sample size, and historical treatment context preclude conclusions regarding additive efficacy beyond contemporary RAS-based supportive care.

### 4.7. Combination with Other Agents

Not all combination strategies have yielded positive results. A multicenter trial enrolling children and young adults that compared omega-3 PUFAs, alternate-day prednisolone, and placebo found no significant benefit on the primary renal outcome over two years [63,72]. However, interpretation was limited by baseline proteinuria imbalances across treatment arms and short follow-up duration. Post-hoc analyses suggested a dose-dependent effect on proteinuria that warrants further evaluation. Furthermore, a pilot uncontrolled trial demonstrated that adding DHA to established EPA monotherapy stabilized renal function over six months; the median eGFR change improved from −7.35% during monotherapy to +1.26% following the addition of DHA, though proteinuria remained unchanged [66]. However, the uncontrolled design and small sample size of these studies preclude definitive conclusions.

### 4.8. Meta-Analytic and Systematic Review Perspectives

Initial meta-analytic efforts reflected the uncertainty of the early evidence base. An analysis of five controlled studies found conflicting results on kidney outcomes, with no statistically significant pooled effect, though a potential benefit was suggested among patients with higher baseline proteinuria [73]. A subsequent evidence-based survey similarly concluded that the pooled RCT data did not support a definitive beneficial effect on renal function [74].

As the evidence base expanded, two analyses converged on a consistent finding. The 2011 Cochrane review of non-immunosuppressive IgAN treatments [70] and a focused meta-analysis of five IgAN-specific RCTs (*n* = 233) [71] both reported a statistically significant reduction in proteinuria, quantified as a weighted mean difference of −0.29 g/day (95% CI: −0.44 to −0.14) in the latter analysis, without a commensurate improvement in GFR. A contemporaneous meta-analysis of 17 omega-3 PUFA trials across heterogeneous kidney conditions similarly reported a significant overall reduction in urine protein excretion [69]; however, the IgAN-specific subgroup was non-significant (Cohen’s d: −0.05; 95% CI: −0.31 to 0.21; *p* = 0.71), with the pooled signal driven principally by mixed-etiology and diabetic nephropathy cohorts. This underscores that the anti-proteinuric effect of omega-3 PUFAs is not uniform across kidney disease diagnoses.

More recent analyses have examined whether early proteinuria reduction translates into protection against hard clinical endpoints. An individual-patient meta-analysis of 11 IgAN randomized trials supported early proteinuria reduction as a surrogate for long-term kidney outcomes. In the fish-oil subgroup, fish oil was associated with a favorable effect on the composite endpoint of serum creatinine doubling, ESRD, or death (HR: 0.44; 95% CI: 0.20 to 0.95) [75]; however, this subgroup finding should be interpreted cautiously because the study was primarily designed to evaluate proteinuria as a surrogate endpoint rather than to provide a definitive estimate of fish-oil efficacy [75]. A complementary meta-analysis of nine RCTs, seven of which focused on IgAN, reported a significant reduction in ESRD risk (RR: 0.49; 95% CI: 0.24 to 0.99) and lower proteinuria (SMD: −0.31; 95% CI: −0.53 to −0.10), with minimal effect on creatinine clearance or eGFR [76]. However, the small number of events, mixed disease populations, and heterogeneity in trial designs limit the certainty and IgAN-specific applicability of these findings.

Despite these signals, more recent systematic reviews continue to emphasize the limitations of the current evidence. A recent systematic review across heterogeneous kidney disease populations, including studies of IgAN, reported no statistically significant overall effect of omega-3 fatty acids on proteinuria and identified substantial heterogeneity across studies [67]. The most current Cochrane review, encompassing 80 studies on non-immunosuppressive treatments for IgAN including fish oil, concluded that fish oil provided only small or non-significant benefits for kidney function compared with placebo or standard care, with low to very low certainty of evidence [68]. Notably, the review identified a significant reduction in the risk of a ≥50% increase in serum creatinine with fish oil (RR: 0.20; 95% CI: 0.06 to 0.65) yet found a paradoxical increase in serum creatinine when fish oil was compared with placebo or no treatment. This finding was reversed when fish oil was compared with active antihypertensive therapies, namely RAS inhibitors, illustrating the critical influence of comparator selection on pooled estimates [68]. Given the small number of events, inconsistent comparator groups, and low to very low certainty of evidence, the apparent reduction in creatinine-doubling risk and the paradoxical serum-creatinine finding do not provide reliable estimates of treatment effect. Overall, these comparator-sensitive findings are best interpreted as evidence of substantial uncertainty rather than evidence for benefit or harm.

In summary, the inconsistency of results across individual RCTs and meta-analyses positions omega-3 PUFAs in IgAN as an experimental/adjunctive approach rather than an established therapy.

### 4.9. Sources of Heterogeneity: Why Results Remain Inconsistent

The marked variability in outcomes across individual trials and meta-analyses can be attributed to several converging factors. At the interventional level, dosing regimens ranged from 0.85 g EPA/0.58 g DHA to 3.76 g EPA/2.94 g DHA daily, with no consensus on the optimal total dose or EPA:DHA ratio. Differences in product source, purity, EPA:DHA ratio, and potentially chemical form may have contributed to variation in biological exposure. However, formulation characteristics were incompletely reported in several historical studies, limiting formal comparisons across trials. Treatment durations have also varied from six months [64] to more than six years [25], and the timing of initiation relative to disease stage may be critical, although the influence of disease stage and chronic fibrosis on omega-3 responsiveness has not been prospectively established.

At the patient level, differences in baseline proteinuria, eGFR at enrollment, concomitant RAS blockade, and the use of immunosuppression further confound cross-trial comparisons. Furthermore, substantial variability exists in the histological lesion profiles of enrolled cohorts. Crucially, most landmark omega-3 trials were conducted prior to the development and widespread clinical adoption of the Oxford Classification (MEST-C score) [77] and the International IgAN Risk Prediction Tool [78]. Consequently, these historical studies lacked standardized, risk-stratified treatment decisions based on quantifiable active inflammation (e.g., endocapillary hypercellularity or crescents) versus irreversible chronic damage, severely limiting the histological comparability of their cohorts. The choice of primary endpoint has also varied substantially, encompassing proteinuria magnitude, eGFR slope, serum creatinine doubling, and composite renal survival outcomes, limiting direct synthesis of findings across studies [67,68,69,70,71]. Lastly, the nature of the control group represents an additional, frequently underappreciated source of variability. As observed in a recent meta-analysis, pooled renal function outcomes fluctuate drastically depending on whether fish oil is strictly paired against an inactive placebo or an active antihypertensive comparator [68]. This divergence underscores that comparator selection remains a critical driver of statistical heterogeneity in pooled estimates.

Taken together, these multidimensional sources of heterogeneity prevent a reliable population-level estimate of treatment effect and preclude confident identification of responders. It is plausible, but unproven, that baseline risk, proteinuria, chronic fibrosis, background therapy, achieved omega-3 exposure, and treatment duration modify response. Future trials should be designed to test these potential effect modifiers prospectively rather than infer them from small historical datasets.

The available evidence does not identify a validated responder population for omega-3 supplementation in IgAN. Historical trials and exploratory analyses have suggested that patients with higher baseline proteinuria or a greater risk of progression may be a candidate population for future investigation. However, these observations are hypothesis-generating, were derived largely from small historical datasets, and have not been prospectively validated in patients receiving contemporary guideline-directed therapy. Therefore, current evidence does not support routine omega-3 supplementation in any defined clinical, histologic, or biomarker-based subgroup. Potential small-study effects and publication bias should also be considered. Much of the evidence base consists of small, single-center studies, several of which reported favorable findings, whereas larger or more rigorously controlled studies have been neutral or inconclusive. Formal assessment of publication bias is limited by the small number of eligible trials and substantial clinical heterogeneity; nevertheless, selective publication and small-study effects may have contributed to the apparent variability of results.

### 4.10. Knowledge Gaps and Priorities for Future Research

The current evidence base leaves several clinically important questions unresolved. First, contemporary randomized trials are needed because most available studies predate current guideline-directed therapy, including maximally tolerated RAS blockade, SGLT2 inhibition, targeted-release budesonide, endothelin-pathway therapies, and complement- or B-cell-directed treatments. Whether omega-3 PUFAs provide incremental benefit when added to these therapies is unknown.

Second, future studies should use biomarker- and risk-guided patient selection. Potential enrichment variables include persistent proteinuria despite optimized supportive care, baseline eGFR slope, Oxford MEST-C score [77], chronicity indices, circulating Gd-IgA1 and complement biomarkers, inflammatory lipid mediator profiles, and erythrocyte omega-3 index. These variables should be evaluated prospectively as potential enrichment factors rather than assumed to identify patients likely to respond to omega-3 supplementation.

Third, intervention protocols should standardize the omega-3 formulation, EPA:DHA ratio, chemical form, purity, dose, and adherence assessment. Measurement of circulating or erythrocyte EPA and DHA levels would help establish exposure–response relationships and distinguish inadequate biological exposure from true treatment non-response.

Finally, trials should be adequately powered and sufficiently long to assess clinically meaningful renal outcomes. Preferred endpoints include chronic eGFR slope, sustained decline in eGFR, kidney failure, and validated composite kidney outcomes, with proteinuria considered an important intermediate endpoint rather than the sole indicator of benefit.

## 5. Conclusions and Perspectives

Omega-3 PUFAs have biologically plausible anti-inflammatory, pro-resolving, and anti-fibrotic effects in experimental systems relevant to IgAN. However, mechanistic plausibility and preclinical findings should not be interpreted as evidence of clinical efficacy. Randomized trials of omega-3 PUFAs in IgAN have produced inconsistent results, with some studies reporting reductions in proteinuria or slower renal function decline and others showing no meaningful benefit in proteinuria, eGFR, or other kidney outcomes.

Meta-analyses suggest possible modest antiproteinuric effects in some settings; however, estimates of kidney-function preservation and kidney-failure prevention remain uncertain because of small sample sizes, heterogeneous patient populations, variable background therapy, differences in formulations and doses, inconsistent endpoints, and limitations in trial quality. Most available trials also predate widespread use of contemporary guideline-directed supportive care, SGLT2 inhibitors, targeted-release budesonide, sparsentan, and complement- or B-cell-directed therapies.

Accordingly, omega-3 PUFAs should not be regarded as established disease-modifying therapy for IgAN or as a replacement for optimized supportive care and approved therapies where indicated.

Future randomized trials should evaluate whether standardized omega-3 formulations provide incremental benefit beyond contemporary background therapy. Such studies should incorporate adequate sample sizes, adherence and exposure assessment, biomarker-informed enrichment strategies, and clinically meaningful outcomes, including chronic eGFR slope, sustained eGFR decline, kidney failure, and validated composite kidney endpoints.

## Figures and Tables

**Table 1:** Omega-3 PUFA (Fish Oil) interventions in IgA nephropathy: Key clinical trials.

First Author, Year	Intervention & Dosage (Daily)	Control Group	Population (*n*)	Follow-Up (Years)	Main Findings
Hamazaki et al., 1984 [58]	EPA (1.6 g) + DHA (1 g)	No treatment	10 vs. 10	1	- Stabilized serum creatinine. - No hemodialysis in treated group (vs. 2 in control). - Suggested possible benefit; small uncontrolled study.
Bennett et al., 1989 [59]	EPA (10 g)	No treatment	17 vs. 20	2	- No benefits on kidney function, despite plasma fatty acid profile changes.
Donadio et al., 1994 [27] Primary trial	Fish oil 12 g/day; EPA 1.68–1.87 g/day and DHA 0.97–1.36 g/day, depending on formulation	Olive oil placebo	55 vs. 51	2	- ≥50% serum creatinine increase: 6% vs. 33% at 2 years. - Proteinuria: no significant between-group difference. - Death or ESRD: 10% vs. 40% at 4 years during extended follow-up.
Pettersson et al., 1994 [26]	EPA (3.3 g) + DHA (1.8 g)	Corn oil placebo	15 vs. 17	0.5	- No significant difference in proteinuria or urinary red blood cell counts. - Slight but significant reduction in GFR in treated group.
Donadio et al., 1999 [25]	Fish oil 12 g/day; EPA 1.68–1.87 g/day and DHA 0.97–1.36 g/day	Olive oil placebo	55 vs. 51	~6.4	- Long-term follow-up: Early and prolonged fish oil treatment slowed renal progression for high-risk IgAN patients.
Donadio et al., 2001 [60]	High dose: EPA (3.76 g) + DHA (2.94 g); Low dose: EPA (1.88 g) + DHA (1.47 g)	Historical controls (placebo group from prior trial)	73 total	2	- High- and low-dose omega-3 fatty acids were similarly effective in slowing renal function loss in high-risk patients. - Fewer patients required dialysis vs. historical controls.
Alexopoulos et al., 2004 [61]	EPA (0.85 g) + DHA (0.57 g)	No treatment	14 vs. 14	4	- Reported slower renal progression (1 treated vs. 6 untreated reached primary endpoint).
Sulikowska et al., 2004 [62]	EPA (0.81 g) + DHA (0.54 g)	No control group (single-arm study)	20	1	- Improved renal vascular and tubule function. - Reduced proteinuria and homocysteine. - No significant change in creatinine clearance.
Hogg et al., 2006 [63]	EPA (1.88 g) + DHA (1.48 g)	Placebo (corn oil) (multi-arm study also included prednisolone)	32 (O3FA) vs. 31 (Placebo) (Total 96 across 3 arms)	2	- No significant benefit on the primary renal outcome in the multi-arm trial. - Post-hoc analysis suggested dose-dependent effect on proteinuria.
Ferraro et al., 2008 [64]	PUFA (3 g total) + RAS blockade	RAS blockade alone	15 vs. 15	0.5	- Omega-3 PUFAs combined with RAS blockade reduced proteinuria and urinary red blood cell count more than RAS blockade alone.
Moriyama et al., 2013 [65]	RAS blockade + EPA (varied dosage: 1.8 g, 1.2 g, or 0.9 g daily)	RAS blockade + dilazep	18 vs. 20	1	- EPA potentially accelerated the anti-proteinuric effects of RAS blockade.
Moriyama et al., 2018 [66]	Switched from EPA (1.8 g) to EPA (1.86 g) + DHA (1.5 g)	EPA alone	21	1 (6 months pre, 6 months post switch)	- Addition of DHA to EPA stabilized renal function; improved %eGFR (-7.35% to 1.26%). - Suggested DHA’s pleiotropic effects stabilize renal function (no significant change in proteinuria).

Abbreviations: DHA: Docosahexaenoic Acid; eGFR: Estimated Glomerular Filtration Rate; EPA: Eicosapentaenoic Acid; IgAN: IgA Nephropathy; O3FA: Omega-3 Fatty Acids; PUFA: Polyunsaturated Fatty Acids; RAS blockade: Renin-Angiotensin System blockade.

## Data Availability

No new data were created or analyzed in this study. Data sharing is not applicable to this article.

## Abbreviations

The following abbreviations are used in this manuscript:

AMPK	AMP-activated protein kinase
BP	Blood pressure
CYP450	Cytochrome P450
DHA	Docosahexaenoic acid
ECM	Extracellular matrix
eGFR	Estimated glomerular filtration rate
EMT	Epithelial–mesenchymal transition
EPA	Eicosapentaenoic acid
FFA4	Free fatty acid receptor 4 (GPR120)
Gd-IgA1	Galactose-deficient IgA1
GPR	G-protein-coupled receptor
IL-6	Interleukin-6
ILK	Integrin-linked kinase
MAPK	Mitogen-activated protein kinase
MCP-1	Monocyte chemoattractant protein-1
MMPs	Matrix metalloproteinases
NF-κB	Nuclear factor kappa B
NLRP3	NLR family pyrin domain-containing
3PPAR	Peroxisome proliferator-activated receptor
RAS	Renin–angiotensin system
Smad3	Mothers against decapentaplegic homolog 3
SPMs	Specialized pro-resolving mediators
TG	Triglycerides
TGF-β1	Transforming growth factor beta 1
TIMPs	Tissue inhibitors of metalloproteinases
TNF-α	Tumor necrosis factor-alpha
TxA	Thromboxane A
